# A HEALTHY LIFE FOR AFRICAN AMERICAN WOMEN CARING FOR OLDER ADULTS: A CONCEPT MAPPING STUDY

**DOI:** 10.1093/geroni/igz038.404

**Published:** 2019-11-08

**Authors:** Abiola O Keller

**Affiliations:** Marquette University, Milwaukee, Wisconsin, United States

## Abstract

Achieving optimal health and well-being among African American older adults with chronic conditions requires addressing the needs of their caregivers. This study aimed to elucidate how African American females caring for older adults view health and the factors that influence health. We identified African American women ages 24 to 64 caring for an adult 60 years or older for group concept mapping (GCM), a mixed-methods approach. Participants (N=25) first completed idea generation by providing unlimited short, free-text responses to the focus prompt, “A healthy life for a caregiver includes: ___.” The 512 identified factors were reduced to 99 unique ideas. Participants then sorted the 99 ideas into clusters based on conceptual similarity and rated each idea on desirability and familiarity. Ratings were recorded on a 5-point Likert scale, ranging from very undesirable to extremely desirable and not at all familiar to extremely familiar. Data were analyzed and mapped via CS Global Max software. A cluster map with 12 outcome domains best fits the data. Identified clusters included: (1) Spirituality, (2) Maintaining relationships, (3) Good character, (4) Action to cope, (5) Preserving self, (6) Support, (7) Personal empowerment, (8) Resources, (9) Release (10) Striving for peace, (11) Wellness, (12) Self-care. Seven of the 99 ideas (representing 5 of 12 domains) were rated as desirable but unfamiliar (“go zone”). We identified elements necessary for health and wellbeing from the perspective of African American caregivers. Go-zone items represent opportunities to intervene to promote the health of African American women caring for older adults.

